# Dynamic evaluation of unruptured intracranial aneurysms by 4D-CT angiography: comparison with digital subtraction angiography (DSA) and surgical findings

**DOI:** 10.1186/s12880-023-01107-1

**Published:** 2023-10-18

**Authors:** Liping Yang, Xing Gao, Chao Gao, Shichuan Xu, Shaodong Cao

**Affiliations:** 1 Department of PET-CT, Harbin Medical University Cancer Hospital, Harbin, China; 2 Department of physical diagnostics, Heilongjiang Provincial Hospital, Harbin, China; 3 Medical Imaging Department, The Fourth Affiliated Hospital of Harbin Medical University, Harbin, 150001 China; 4 Department of medical instruments, Second Hospital of Harbin, Harbin, 150001 China

**Keywords:** Four-dimensional CT angiography (4D-CTA), Unruptured intracranial aneurysms, Digital subtraction angiography (DSA), Aneurysm pulsation

## Abstract

**Background:**

This study was to prospectively investigate the feasibility of four-dimensional computed tomography angiography (4D-CTA) with electrocardiogram-gated (ECG) reconstruction for preoperative evaluation of morphological parameters, and compared with digital subtraction angiography (DSA). We also aimed to detect pulsation in unruptured intracranial aneurysms (UIAs) by using 4D-CTA, as a potential predicting factor of growth or rupture.

**Materials:**

64 patients with 64 UIAs underwent ECG-gated dynamic 4D-CTA imaging before treatment, of which 46 patients additionally underwent DSA. Original scanning data were reconstructed to produce 20 data sets of cardiac cycles with 5%-time intervals. The extent of agreement on UIAs morphological features assessed with 4D-CTA and DSA was estimated using the k coefficient of the Kappa test. The radiation doses were also calculated and compared between 4D-CTA and DSA. In the aneurysmal surgically treated in our institution, we were able to compare the surgical findings of the aneurysm wall with 4D-CTA images. We performed long-term follow-up on untreated patients.

**Results:**

The morphological characteristics detected by 4D-CTA and DSA were consistent in aneurysm location (k = 1.0), shape (k = 0.76), maximum diameter (k = 0.94), aneurysm neck (k = 0.79) and proximity to parent and branch vessels (k = 0.85). 4D-CTA required lower radiation doses (0.32 ± 0.11 mSv) than DSA (0.84 ± 0.37 mSv, *P* < 0.001). Pulsation was detected in 26 of the 64 unruptured aneurysms, and all underwent neurosurgical clipping or interventional embolization. In aneurysms surgically treated in our hospital, we observed a significant correlation between 4D-CTA findings and surgical evaluation of the aneurysmal wall, in particular the irregular pulsations detected on 4D-CTA have demonstrated to correspond to dark-reddish thinner wall at surgery.

**Conclusions:**

In this proof-of-concept study, 4D-CTA provided real-time, non-invasive preoperative assessments of UIAs comparable to DSA. Moreover, optimal correlation between the irregular pulsation detected by 4D-CTA and the surgical findings support a possible role of this technique to identify aneurysms with a higher risk of rupture.

**Supplementary Information:**

The online version contains supplementary material available at 10.1186/s12880-023-01107-1.

## Introduction

The incidence rate of cerebral aneurysms is approximately 1-5% in the adult population, which is further complicated by the rupture with permanent neurological deterioration and silent embolic events [1]. In particular, subarachnoid hemorrhage (SAH) from intracranial aneurysm rupture tends to be associated with high rates of mortality and morbidity [2]. Conventional morphological parameters, such as aneurysmal shape, aneurysmal location, and size, are associated with aneurysm rupture; however, these parameters are confounding and controversial [3]. Most researchers believe that the larger the aneurysm, the greater the risk of rupture [4, 5]. However, a series of studies have shown that small aneurysms also have a high possibility of rupture [6]. Morphology has provided an important basis on which to study the risk of aneurysm rupture, but most computed tomography angiography (CTA) studies of aneurysm morphology have been based on static data generated by three-dimensional CTA (3D-CTA) [7]. It may be more useful to dynamically investigate the morphological changes of intracranial aneurysms. Exactly, the pulsation point in a cardiac cycle is a morphological indicator that reflects the dynamic change of an aneurysm at a weak point in the aneurysm wall [8].

Digital subtraction angiography (DSA) provides a relatively high spatial and temporal resolution and is the current “gold standard” for the evaluation of intracranial aneurysms, but it is an invasive, expensive, and time-consuming process [9]. In contradistinction, CTA is a minimally invasive, fast, and simple procedure to assess the anatomic details of UIAs [10]. Moreover, CT technology is rapidly advancing with the introduction of a 320-row CT scanner that can acquire volumetric 3D-CTA scans of the whole brain in a sub-second time [11]. Combined with ECG-gating, 320-row CT scanners can now perform dynamic studies of UIA over a cardiac cycle, so-called four-dimensional CTA (4D-CTA). Unlike 3D-CTA, 4D-CTA can acquire time-resolved three-dimensional reconstructions to provides dynamic information on aneurysm wall and visualizes wall motion during cardiac cycle. Therefore, irregular pulsation detected by 4D-CTA could be a novel imaging marker of aneurysm vulnerability [12]. The objective of this proof-of-concept study is to investigate the association of the morphological risk factors characterized by 4D-CTA and DSA. With this prospective observational study, we also aimed to visualize aneurysm irregular pulsation by means of ECG-gated 4D-CTA in a series of unruptured intracranial aneurysms, investigating the correlations with intraoperative findings in a subset of patients who underwent neurosurgery.

## Materials and methods

This prospective study was reviewed, approved, and overseen by the institutional review board of The Fourth Affiliated Hospital of Harbin Medical University and conducted under the 1964 Declaration of Helsinki and its later amendments or comparable ethical standards. All subjects signed informed consent forms in line with the local ethics committee’s regulations for prospective research.

### Patients

**Fig. 1:**
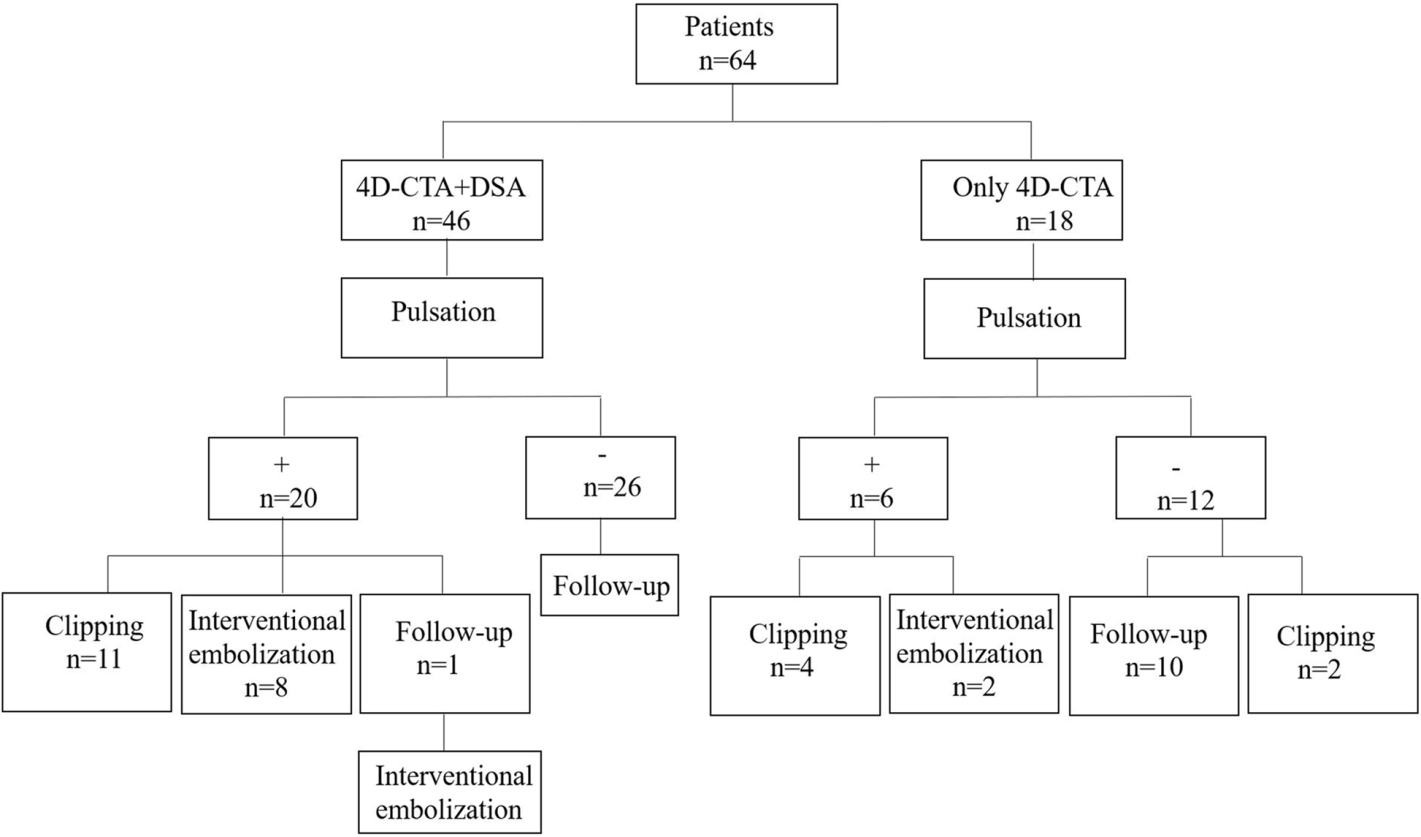
Diagnostic and therapeutic outcomes in patients with UIAs

 This was a prospective study on 64 consecutive patients with 64 UIAs who underwent 4D-CTA and were enrolled from July 2016 to June 2018. 4D-CTA is a routine examination for patients with intracranial aneurysms in our hospital. Among them, 46 patients also underwent DSA examination (Fig. 1). Clinical information, including sex, age, family history, smoking history, and hypertension was prospectively collected. Inclusion criteria are the following: 1) patients between 18 and 80 years of age; 2) all 4D-CTA images demonstrated a clear edge of the aneurysm, and conform to the diagnostic criteria; 3) patients or their families gave written informed consent. Exclusion criteria are the following:1) cerebral aneurysms with a high risk of rupture (multiple aneurysms, history of subarachnoid hemorrhage); 2) secondary intracranial cerebral aneurysm (aneurysms caused by infection or trauma); 3) history of cerebrovascular malformations; 4) dissecting aneurysms; 5) women who are pregnant or breastfeeding and 6) patients with severe systemic diseases such as acute myocardial infarction, renal dysfunction, and malignant tumors.

### 4D-CTA image acquisition, interpretation and analysis

Retrospective ECG-gated CTA was performed with the volumetric imaging mode using a 320-row CT scanner (Acquilion ONE, Toshiba medical systems, Japan). Scan parameters were: 120-KV tube voltage, 270-mA tube current, 350ms gantry rotation time, and 160 mm Z-coverage. The field of view (FOV) was 240 mm with an imaging matrix size of 512 × 512. First, a non-enhanced helical CT head acquisition was performed (120 kV, mA modulation). Then, a low-dose pre-contrast volume scan was performed as a subtraction mask. Subsequently, nonionic contrast medium (100mL, Iopamiron 370; Schering, Osaka, Japan) was injected intravenously at a rate of 3 mL/s 20–23 s post-injection or in conjunction with the CT scanner bolus-tracking function (SureStart; Toshiba).

4D-CTA source data were transferred to an image processing workstation (Vitrea 2 software 6.02, Toshiba, Japan) for ECG-gated reconstruction with the cardiac cycle (i.e., R-R interval) divided into 20 phases (from 0 to 95% every 5% cardiac circle). All phases were reconstructed with slice thickness of 0.25 mm and 0.25 mm interval. In this study, the vessel wall in the 3D or 4D image was determined by the aneurysm cavity, and a voxel threshold greater than 200HU was determined as an aneurysm cavity containing contrast agents. Two experienced neuroradiology readers, blinded to the DSA results, independently evaluated the 4D-CTA images for UIA location, shape, maximum diameter, the neck of the aneurysm, and proximity to parent and branch vessels. In the rare case of discordant assessments, the images were reassessed to reach a consensus.

### ECG-gated reconstruction method

ECG-gated reconstruction is a method for minimizing cardiac motion artifacts by performing image acquisition while monitoring the ECG [13]. Recently, with the increase of helical CT tube rotation speed and the introduction of multisession technology, it is possible to apply the ECG-gated reconstruction method to minimize cardiac motion artifacts in imaging of the coronary arteries and the aortic arch [14]. The half-reconstruction method and the segmental reconstruction method are two different ECG-gated reconstruction methods. The former method can improve the temporal resolution regardless of the beam pitch. The latter method can also be used to improve the temporal resolution, but it suffers from limitations in terms of helical pitch, tube rotation speed, and heart rate [15]. Our study adopts a combination of half-reconstruction and segmental reconstruction to improve the temporal resolution. Specifically, one cardiac cycle was divided into 20 phases, and the images were processed.

### Criteria for identifying aneurysmal pulsation

**Fig. 2:**
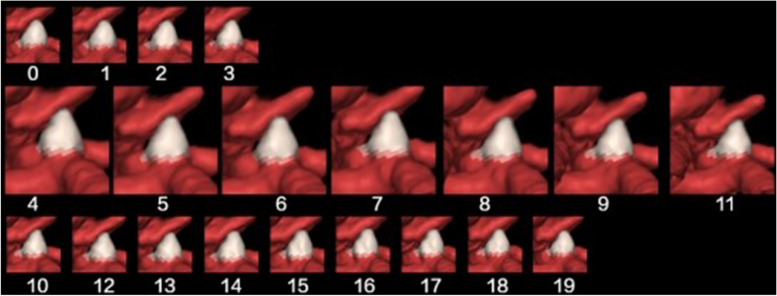
Criteria for identifying aneurysmal pulsation: Twenty frames corresponding to the twenty phases in the R-R interval of a single cardiac cycle were acquired. Elevation in a part of the aneurysm was observed in at least three successive frames

The RR interval was divided into 20 phases at 5% intervals for ECG-gated reconstruction, and aneurysmal pulsation was determined to be present when a small bleb-shaped protuberance in a part of the aneurysm was observed in at least 3 consecutive images (Fig. 2). The decision of whether or not a pulsation was present was made by one neurosurgeon and one radiologist, respectively. All of them carefully reviewed the 4D-CTA data of the aneurysms as a group. In the case of different judgments between the two reviewers, a third more experienced neuroradiologist (with more than 15-year experience) was consulted for final decision. The detection of aneurysmal pulsation was displayed as movies (see Supplementary Materials).

### Digital Subtraction angiography

DSA was performed using Axiom Artis Unit System (Siemens, Germany). A standard four-vessel study was performed with a 3D rotational sequence for enhanced delineation of the aneurysms. UIA morphological features, including location, shape, maximum diameter, the neck of the aneurysm, and proximity to parent and branch vessels, were assessed with DSA as performed for CTA.

### Effective radiation dose estimation

Radiation exposure was determined for each technique. The dose-length product (DLP) values of DSA and 4D-CTA and the total volume CT dose index (CTDI vol) of 4D-CTA were determined. The effective dose was determined as the DLP value multiplied by a conversion factor of 0.021 mSV/mGy•cm.

### Post-operative evaluation of 4D-CTA and outcome follow-up

Neurosurgeons post-operatively graded the preoperative value of 4D-CTA information for the surgical intervention: grade 1 was defined as highly useful and sufficient for surgical treatment decisions, grade 2 as a useful result with insufficient information, and grade 3 was insufficient UIA information requiring additional DSA examination. Patients untreated for UIA were contacted and interviewed for clinical neurological status. In addition, these patients underwent a first follow-up after 6–12 months (CTA or magnetic resonance angiography examination), followed by a follow-up every year or every 2 years according to current US guidelines.

### Statistical analysis

Cohen’s kappa coefficient (k) was used to evaluate interpretation consistency between the 4D-CTA and DSA results for each morphologic parameter. Inter-modality consistency was classified as: excellent (k>0.80), good (k = 0.61–0.80); moderate (k = 0.21–0.40), fair (k = 0.21–0.40), or poor (k < 0.20).

## Results

### Baseline characteristics

 Sixty-four unruptured aneurysms from 64 patients (20 males, 44 females; 47.0 ± 12.7 years old) were analyzed by 4D-CTA in order to evaluate the morphological and dynamic features of these lesions. The aneurysms’ medium size at the moment of initial diagnosis was 6.21 ± 3.04 mm and locations were variable. Characteristics of patients and aneurysms are presented in Table 1 along with clinical presentations provided in Table 2.

**Table 1:** Characteristics of patients and aneurysms

Parameters	
Gender of patients (no. of males)	24
Average age of patients (year): mean± SD	47 ± 12.7
Family history (no. of patients)	6
History of smoking (no. of patients)	34
Hypertension (no. of patients)	42
Size of aneurysm (mm): mean± SD	6.21 ± 3.04

*No* Number; *SD* Standard deviation

**Table 2:** Clinical presentations of patients

Symptoms	No. of patients (%)
Headache	26 (40%)
Dizziness	14 (22%)
Aphasis	8 (12%)
Hemiplegia	5 (8%)
Vision changes	6 (9%)
Incidental	5 (9%)

### Inter-modality consistency

 The location of the aneurysm demonstrated in CTA and DSA was presented in Table 3. The aneurysmal shape, maximum diameter, neck, and proximity to parent and branch vessels demonstrated in CTA and DSA were summarized in Table 4. As shown in Table 5, inter-modality, i.e. CTA vs. DSA, agreement in the assessment of aneurysmal location is excellent (k = 1.0; 95% CI 1.00–1.00), in the assessment of aneurysmal shape is good (k = 0.76; 95% CI 0.53–0.89), in the assessment of aneurysmal maximum diameter is excellent (k = 0.94; 95% CI 0.92-1.00), in the assessment of aneurysmal neck is good (k = 0.79; 95% CI 0.54–0.94), in the assessment of proximity to parent and branch vessels is excellent (k = 0.85; 95% CI 0.69-1.00).

**Table 3:** Frequency of location of UIAs in 4D-CTA

				No. of patients (%)	
	CTA		CTAa	DSA	Agreement between CTA and DSA
Aneurysmal location	Reader 1	Reader 2			
MCA-M1	8	8	8	8	46/46(100%)
MCA-M2	6	6	6	6	
AccomA	10	10	10	10	
PcomA	8	8	8	8	
Distal ACA	2	2	2	2	
IC-PC	6	6	6	6	
IC-AChA	4	4	4	4	
Basilar tip	2	2	2	2	
Total	46	46	46	46	

*4D-CTA* Four-dimensional CT angiography; *UIAs* Unruptured intracranial aneurysms; *No. of patients* The number of patients; *CTAa* Interpretations of both readers for CTA were identical, *MCA-MI* The M1 portion of the middle cerebral; *MCA-M2* The M2 portion of the middle cerebral; *AcomA* The anterior communicating artery; *PcomA* The posterior communicating artery; *Distal ACA* The distal anterior cerebral artery; *IC-PC* The internal carotid artery-posterior communicating artery; *IC-AChA* The internal carotid artery-anterior choroidal artery

**Table 4:** Other characteristics of UIAs in 4D-CTA and DSA

		No. of patients (%)				
		CTA		CTAa	DSA	Agreement between CTA and DSA
Parameters	Definition	Reader 1	Reader 2			
Shape	No lobe	22	18	22	14	34/46(74%)
	One or two lobes	16	22	16	18	
	More than two lobes	8	6	8	14	
Maximum diameter	Small (4mm-6mm)	6	6	6	6	42/46(91%)
	Medium (7mm-10mm)	16	16	16	18	
	Large (10mm-25mm)	20	20	20	18	
	Narrow (smaller than the dome diameter)	16	14	16	14	
Neck	Medium (equal to the dome diameter)	20	24	20	26	36/46(78%)
	Wide (larger than the dome diameter)	10	8	10	6	
Proximity to parent and branch vessels	Poor (cannot display parent and branch vessels)	0	0	0	0	40/46 (87%)
	Fair (clear parent but unclear branch vessels)	4	6	4	2	
	Good (clear visualization of parent and branch vessels)	42	40	42	44	

*CTAa* Interpretations of both readers for CTA were identical

**Table 5:** Inter-modality agreement between UIA morphological features assessed with 4D-CTA and DSA using the Kappa test

	Location	Shape	Maximum diameter	Neck	Proximity to parent and branch vessels
Inter-modality agreement	1.00	0.762	0.938	0.794	0.847
	(1.000-1.000)	(0.531-0.889)	(0.920-1.000)	(0.541-0.938)	(0.689-1.000)

Inter-modality: 4D-CTA and DSA

### Pulsation of aneurysm and surgery

Pulsation was detected in 26 of 64 unruptured aneurysm patients with the above criterion. Twenty-six patients with abnormal pulsation detected by 4D-CTA were treated as follows: 15 patients underwent neurosurgical clipping, 10 patients received interventional embolization therapy directly, and 1 accepted interventional embolization therapy because of subarachnoid hemorrhage upon 36 d follow-up. Intraoperative results showed that these dangerous pulsations detected on 4D-CTA were corresponding to the dark-reddish thin walls at the surgery.

### Estimated effective radiation dose

The average DLP, CTDI vol, and effective doses for 4D-CTA were 121.53 ± 14.91 mGy·cm, 11.92 ± 2.14 mGy, and 0.32 ± 0.11 mSv, respectively. The effective dose of 4D-CTA is significantly lower than that of DSA (0.84 ± 0.37 mSv, *P* < 0.05).

### Post-operative evaluation and follow-up

During a post-operative evaluation, neurosurgeons agreed that 4D-CTA could provide enough morphological information for the surgical intervention, including aneurysmal location, shape, maximum diameter, aneurysm neck, and proximity to parent and branch vessels, as well as dynamically monitor the pulsation of UIAs to evaluate its stability in all cases, which is highly useful and sufficient for surgical treatment decision. Additionally, patients untreated for UIAs had no adverse cerebrovascular events during follow-up.

## Discussion

In this study, 4D-CTA was used prospectively to evaluate patients with UIAs and referenced to DSA, and in cases suspected of UIA instability, compared to the surgical presentation of the aneurysm. The overarching experimental conclusion was that 4D-CTA was reliable for the localization and anatomical characterization of UIAs with excellent agreement with DSA results and surgical findings. Importantly, high-resolution temporal acquisitions of UIA pulsation helped to recognize unstable smaller lesions that might be overlooked with static DSA imaging. Advantages of 4D-CTA over DSA included being a non-invasive and lower-cost technique that offered rapid multi-vessels, multi-angle imaging significantly lower effective radiation dose exposure [16, 17].

DSA is the current “gold standard” for the diagnosis of intracranial aneurysms and surgical strategy guide because of its ability to elucidate the UIA pedicle and origin as well as its spatial relationship with nearby vascular branches. The inherent drawbacks of DSA remain potential procedural complications and high costs associated with the invasive intravascular intervention [18]. Magnetic resonance angiography (MRA) is also challenging the routine use of DSA. The diagnostic advantages of MRA for UIA include its non-invasive nature, avoidance of contrast agents, avoidance of radiation, better soft tissue definition, and multi-directional reconstruction and display to enhance assessment of anatomical detail [19, 20]. UIA greater than 3 mm in diameter can be resolved by MRA with an accurate rate of 67–86% [21, 22].

3D-CTA has also evolved to challenge DSA as the reference standard. 3D-CTA is very rapid, low cost, and minimally invasive but does require contrast agents [19]. Improvements for single-slice spiral CT to 16 multi-slice CT have enhanced the detection of cerebral vascular branch vessels to 3–4 branches and increased UIA diagnostic sensitivity to aneurysms larger than 3 mm, similar to MRA [23]. Further development of 64-multislice spiral CT allowed the use of isotropic voxels, such that the reconstructed image achieves the same resolution within or through plane, and in any oblique cross-section. The improved angiography images were further refined through the continual development of more powerful reconstruction and post-processing software [24], which allowed 3D-CTA can make to achieve the same sensitivity, specificity, accuracy, and precision for noninvasive UIA evaluations as DSA [25].

Although aneurysms less than 3 mm have been considered to be the lower limit of detection for 3D-CTA, in the present study, using 320-multi-slice spiral CT, all aneurysms were well recognized on the CTA image including those less than 3 mm [26]. In general, many studies have reported that artifacts, the amount of contrast agent, and CT scan parameters greatly influenced and could detract from CT image quality. However, such artifacts have been minimized with new-generation scanners, such as in the present study, which employed advanced wide-detector CT scanning technology, which facilitated image post-processing results, morphological detail and quantitative measurements [27]. Moreover, the recent implementation of iterative reconstruction algorithms (i.e., maximum likelihood approaches) rather than traditional filtered back projection methods, as used in the present study can further improve image quality and reduce patient radiation exposure, even beyond the significant benefits achieved in this study over DSA [28]. Encouraging, in our experience, CTA has excellent consistency with DSA for the assessment of intracranial unruptured aneurysms. We found that CTA has a high detection rate in aneurysms, which may be due to the removal of bone structure interference by CTA, leading to a higher vascular resolution and increased detection rate of intracranial aneurysms.

Although noninvasive MRA and 3D-CTA enhance the details of UIA and surrounding anatomy, these methods lack the temporal resolution to characterize the dynamics of the aneurysms [29]. Aneurysm features are not always static within the cardiac cycle. They can undergo continuous morphological and volumetric changes that manifest as abnormal pulsation [30], as confirmed in the present study. Large UIA is more likely to rupture than smaller counterparts [31]. However, small aneurysms are also at risk of rupture [32], and discrimination of a small stable UIA versus one at higher risk of rupture is critical to patient risk-benefit stratification for therapeutic intervention. Increasingly, the use of static morphological parameters is questioned [33], with the adjunctive presence of UIA pulsation perhaps being critical to rupture risk decisions, particularly for small UIA [34]. However, while UIA pulsation is suggested to be important in the risk assessment of UIA, the requirements for CT imaging equipment capable of evaluating pulsation have been a practical clinical barrier [35]. While the development of 64-multi-slice spiral CT has found success in overcoming large-scale motions of the heart and the great vessels, requisite long scan time, small z-axis coverage, and low temporal resolution preclude capturing the needed CT scan data within a single cardiac cycle, which is required to observe the minute pulsation of UIAs [10].

**Fig. 3:**
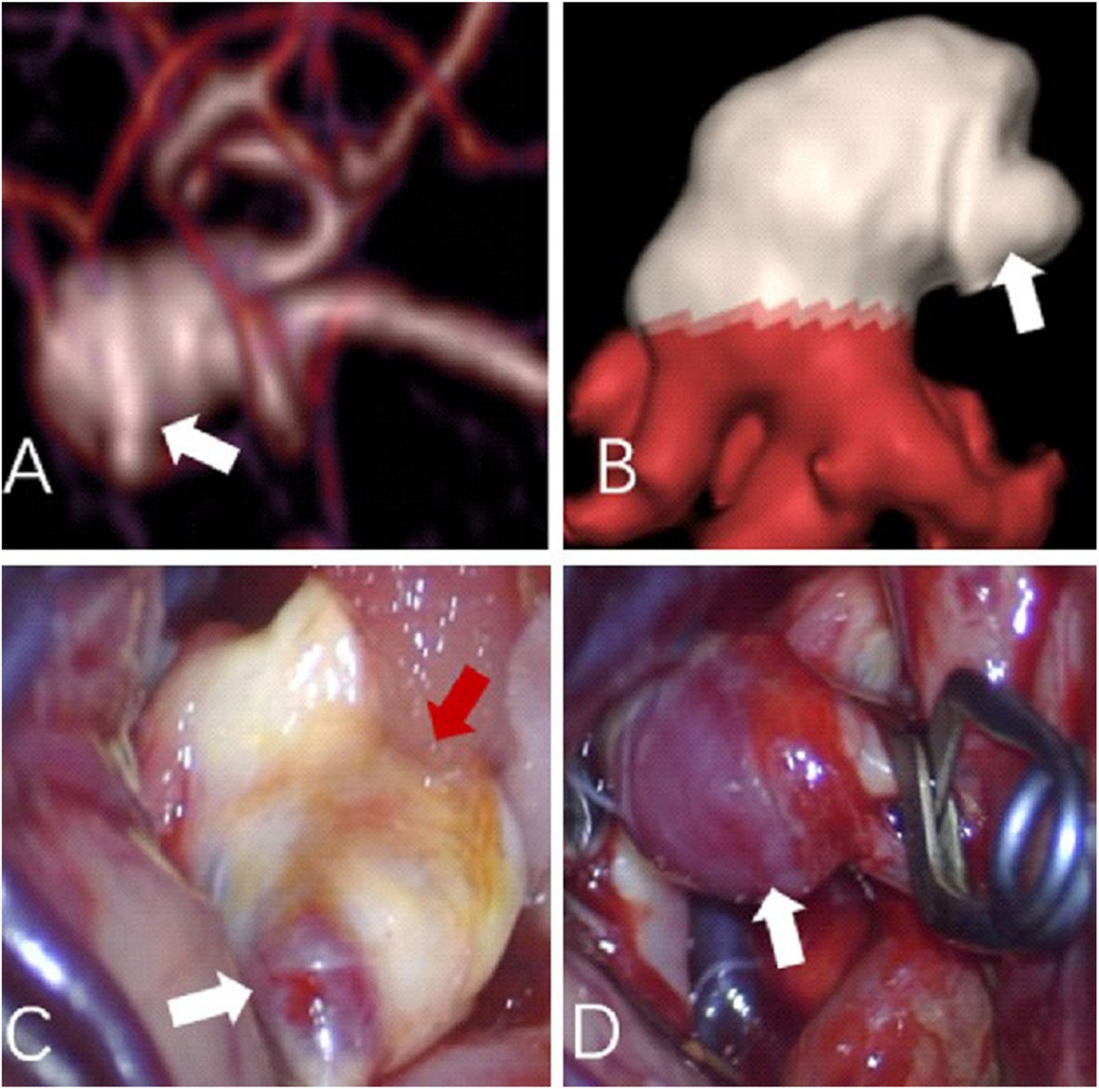
**A**-**B** CTA showed a left MCA-M1 aneurysm (10.8 mm x 5.7 mm), and an obvious pulsation was observed by 4D CTA (as indicated by the white arrow). **C**-**D** Photographs obtained during surgery, small vesicles shaped convex and calcified plaque were seen intra-operation (as indicated by the white and red arrow, respectively), an intra-operational finding confirmed that the abnormal pulsation site is an at-risk aneurysmal wall

**Fig. 4:**
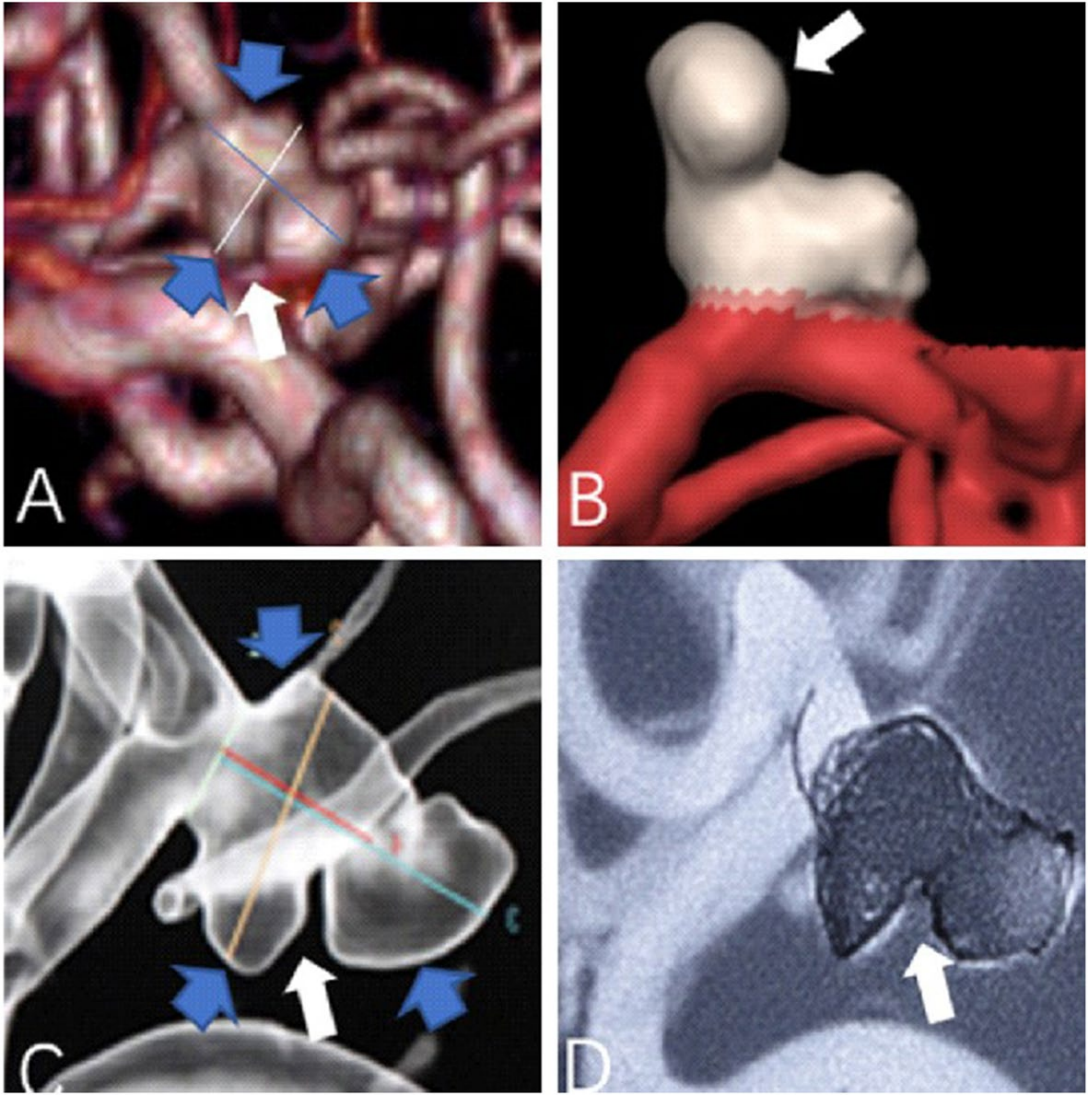
**A**-**B** CTA showed a left posterior communicating artery aneurysm (PcomA) (10.2 mm x 7.8 mm), and an obvious pulsation was observed by 4D-CTA (as shown by the white arrow). Both observers reported a large aneurysm, having more than two lobes (as shown by the blue arrow). a narrow neck, and clear visualization of parent and branch vessels. **C**-**D** DSA findings confirmed CTA findings, the patient underwent interventional embolization therapy directly, the therapeutic effect was shown in frame **D**

**Fig. 5:**
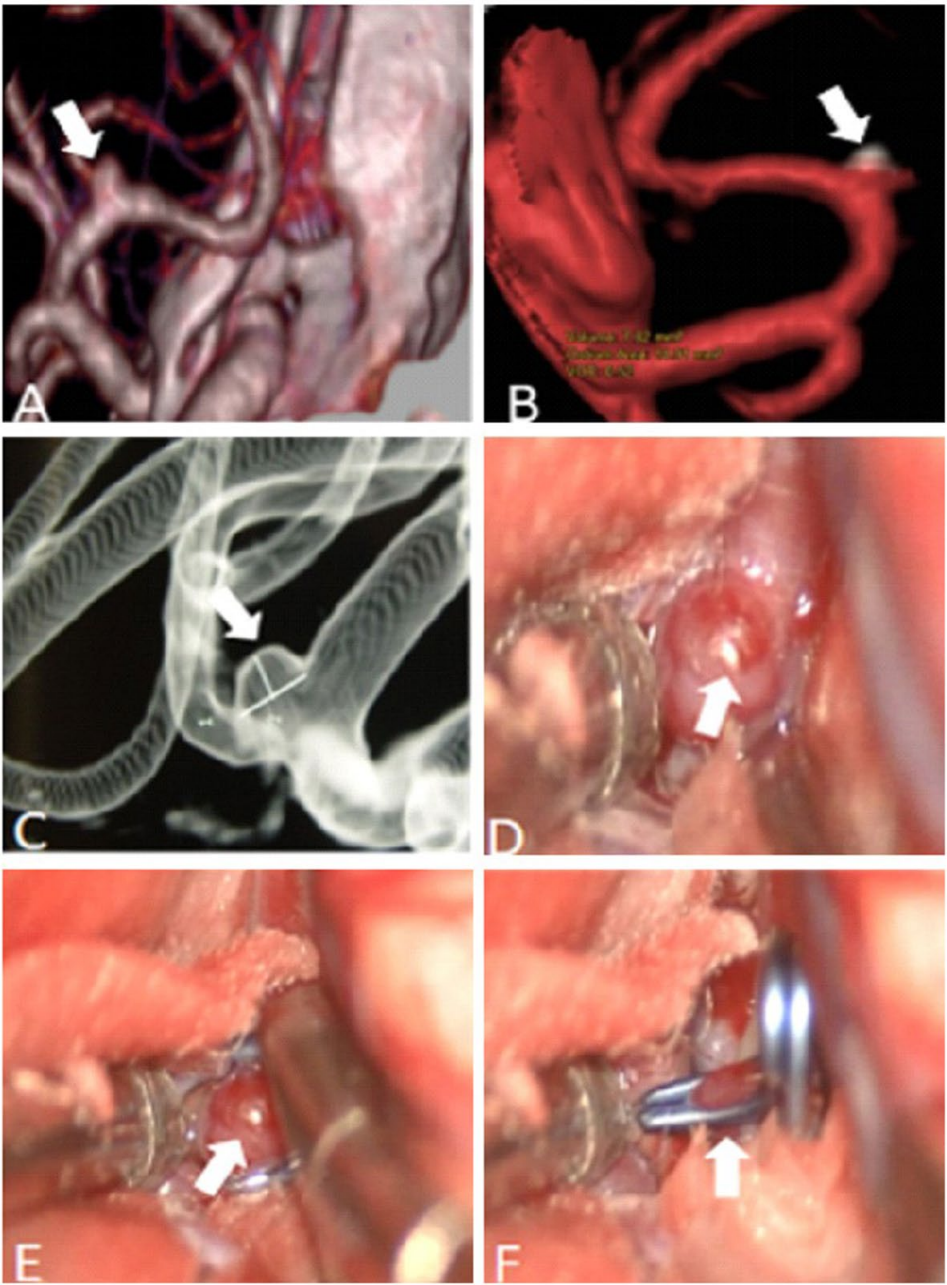
**A**-**B** CTA showed a right MCA-M1 aneurysm (1.5 mm x2.0 mm), and a tiny pulsation was observed by 4D-CTA (indicated by the white arrow). Both observers recorded the aneurysm as small, having no lobe, a wide neck, and clear visualization of parent and branch vessels. **C** DSA findings confirmed CTA findings. **D**-**F** photographs obtained during surgery, an abnormal thin wall of the aneurysm was observed during the surgery, i.e., there was a small protuberance, like a “bleeding blister”. The pulsation site displayed on CTA images colocalized with the relatively weak aneurysmal wall that is prone to rupture

**Fig. 6:**
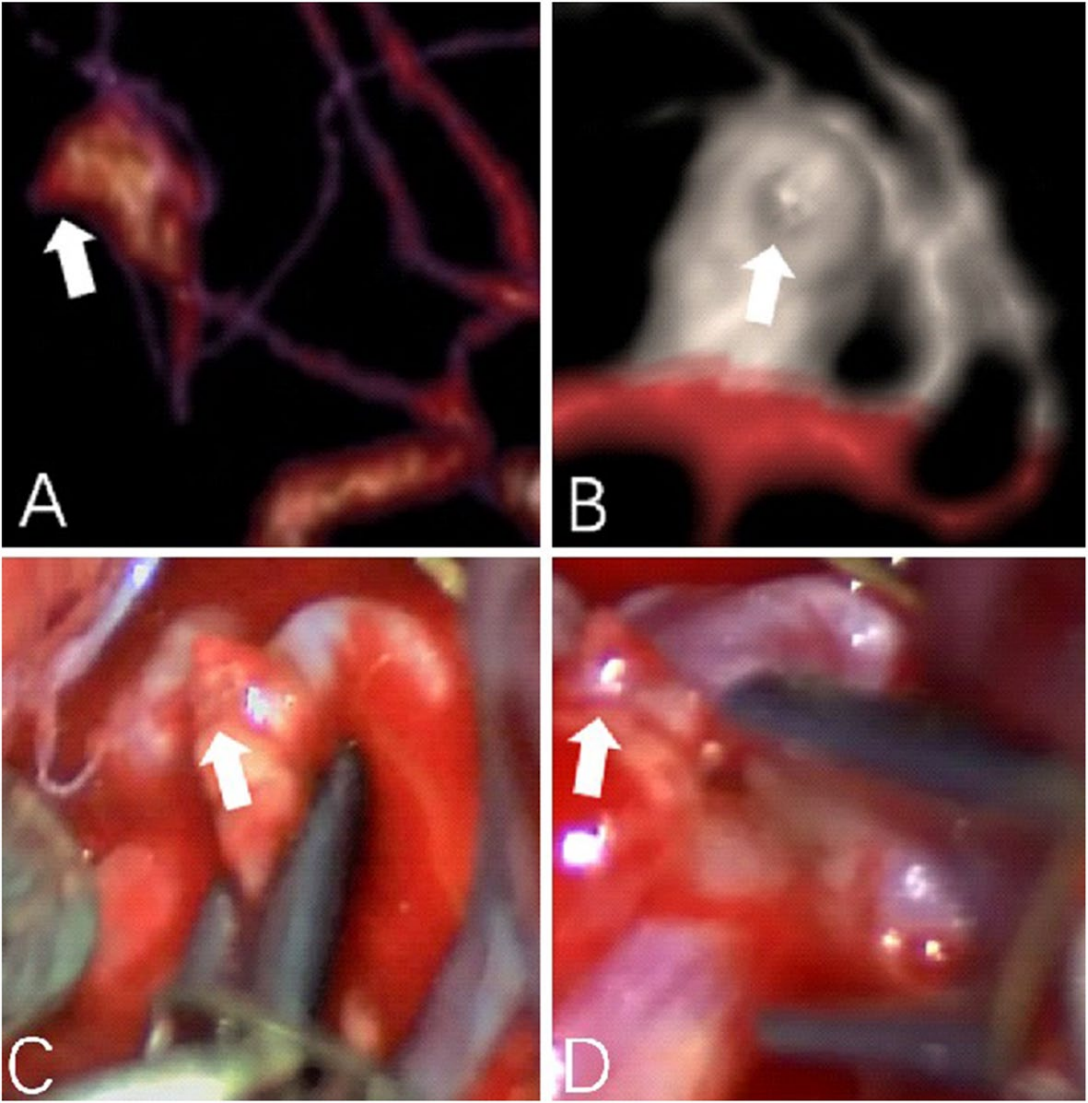
**A**-**B** CTA showed a right MCA-M1 aneurysm (7.6 mm x 5.1 mm), and a tiny pulsation was observed by 4D-CTA (indicated by the white arrow. **C**-**D** photographs obtained during surgery, small vesicles shaped convex were seen intra-operation (as indicated by the white arrow), intra-operational findings confirmed that the abnormal pulsation site is an at-risk aneurysmal wall

In the present study using 320-multi-slice CT to perform 4D-CTA imaging, the 16 cm scan range permitted the field-of-view (FOV) to encompass the entire brain in one rotation of the X-ray tubes without table repositioning of the patient. The improved image quality and temporal resolution were substantial, which was reflected in the definitive detection of abnormal aneurysm pulsation that was subsequently confirmed intraoperatively [36]. In our study, all large cerebral aneurysms in the 4D-CTA imaging showed a significant pulsation, the patients received surgical treatment in time, successfully carried out an aneurysm clamping, effectively prevented and avoided the rupture of the aneurysms, greatly improved the patient’s clinical prognosis. The results of the operation confirmed that the abnormal pulsation site observed in 4D-CTA was corresponding to the weak area of the aneurysmal wall in surgery (as shown in Figs. 3 and 4). In addition, in this study, some of the relatively small cerebral aneurysms appeared stable, but in the 4D-CTA imaging, there also was an abnormal pulsation, the majority of patients actively engaged in craniotomy surgery or interventional embolization treatment, achieved good clinical results and avoided the occurrence of bad cerebrovascular events. Thus, Small and large aneurysms with foreboding pulsations were corroborated to be dark-reddish, thin-wall, high-risk lesions at surgery (as shown in Figs. 5 and 6). Aneurysm irregularity may reflect the focal weakness of the wall of the aneurysm, which is related to degeneration. These changes may lead to a variety of aneurysm wall remodeling, including endothelial cell damage, smooth muscle cell migration and inflammatory cell infiltration. Irregular pulsation will more likely develop, but underlying mechanisms require further studies [37].

**Fig. 7:**
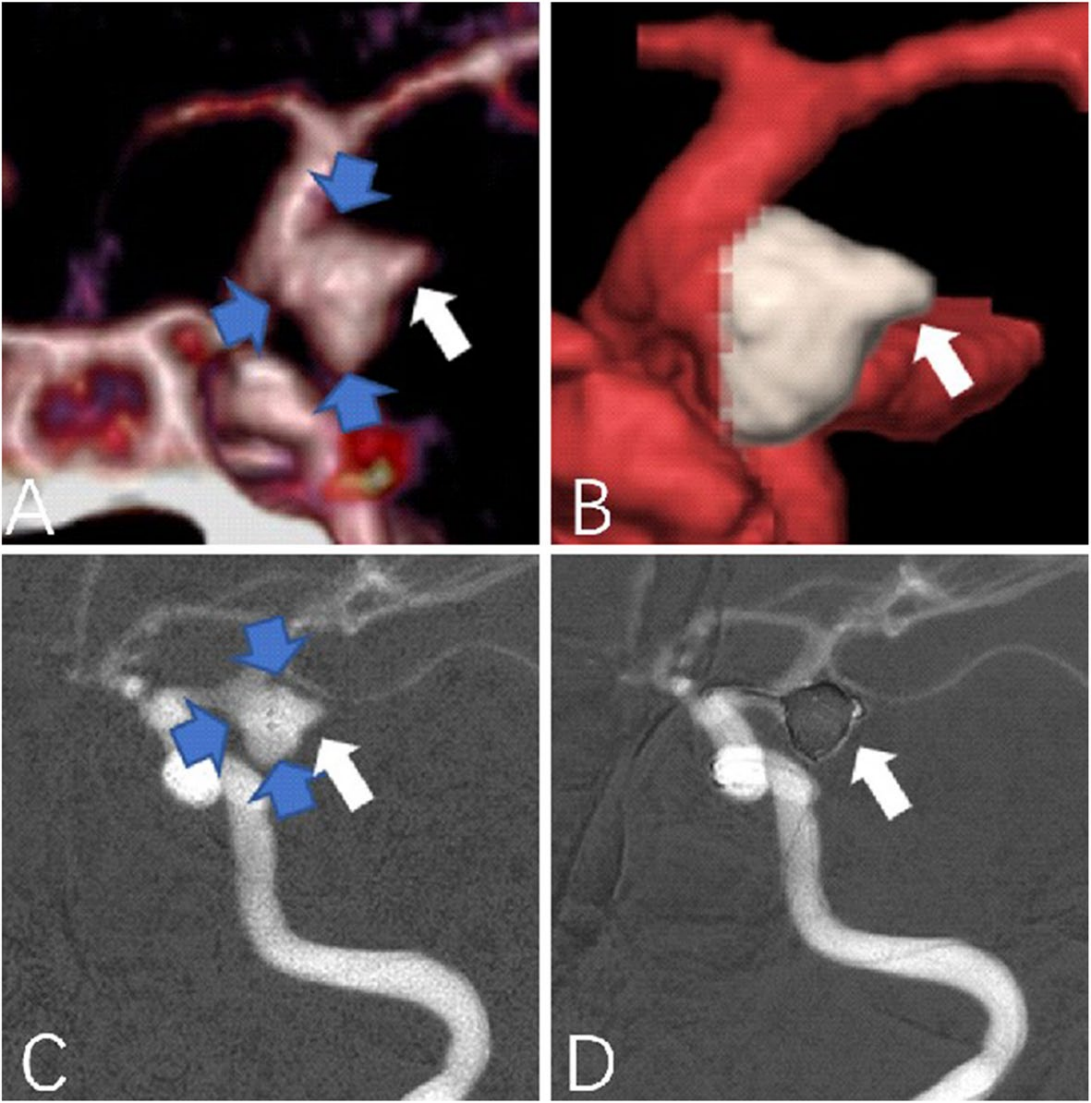
**A**-**B** CTA showed a right posterior communicating artery aneurysm (PcomA) aneurysm (5.5 mm x3.8 mm), and an obvious pulsation was observed by 4D-CTA (as shown by the white arrow). Both observers recorded the aneurysm as small, having more than two lobes (as shown by the blue arrow), a narrow neck, and clear visualization of parent and branch vessels. **C**-**D** DSA findings confirmed CTA findings, the patient underwent interventional embolization therapy after a 36-day follow-up finally in frame **D**

**Fig. 8:**
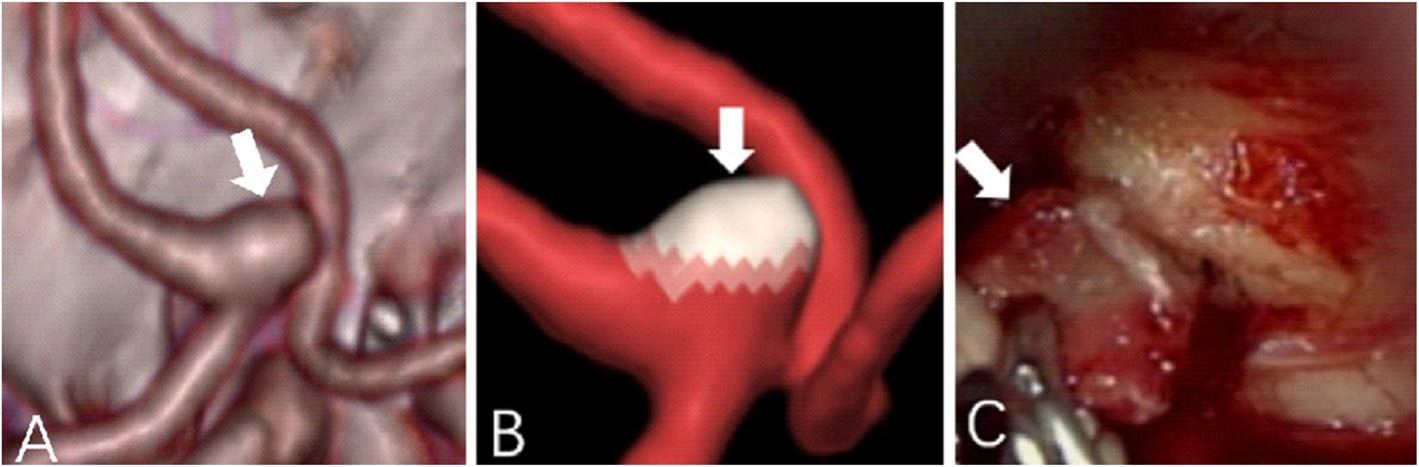
**A**-**B** CTA showed an anterior communicating artery aneurysm (AcomA) (4.2 mm x 3.5 mm), and no obvious pulsation was observed by 4D-CTA (as indicated by the white arrow). **C** Photographs obtained during surgery; a homogenous aneurysmal wall was observed during the surgery (as indicated by the white arrow)

Unfortunately, for one patient with abnormal aneurysmal pulsation detected by 4D-CTA, surgical intervention was deferred. However, 36 days later interventional embolization of a subarachnoid hemorrhage was required when the patient presented severe neurological symptoms (as shown in Fig. 7) was performed. It indicated that the pulsation of the aneurysm was a predictor of the risk of rupture of an aneurysm. In practice, when an abnormal pulsation was found in an unruptured intracranial aneurysm, it indicated a higher risk of rupture, suggesting that the neurosurgeon should take treatment as early as possible to avoid sudden rupture during follow-up. In contrast, the patients without UIA pulsations observed by 4D-CTA were discharged without intervention, and clinical neurological evidence of aneurysm ruptures was reported at the standard regular follow-up contact. We boldly believed that these aneurysms were in a relatively stable state, and currently, there is no occurrence of aneurysmal rupture in those patients. However, long-term follow-up is still required. Two patients without pulsation noted by 4D-CTA voluntarily requested neurosurgical clipping for prevention therapy due to concerns about their own severe hypertension. Intraoperative findings showed more stable homogenous walls (as shown in Fig. 8). To summarize, using 4D-CTA images, we found that irregular pulsation was closely associated with aneurysm rupture, and the discovery of the pulsation with unruptured aneurysms has important implications for the guiding treatment of the aneurysms. This finding is in line with those of some studies that reported irregular pulsation was correlated with the status of aneurysm rupture [10, 38]. Kato et al. observed that aneurysms with irregular pulsation had varied thicknesses and an absence of the internal elastic lamina and tunica media [17]. Krings et al. demonstrated that the presence of abnormal pulsation was related to aneurysm growth [39]. Similarly, Ferrari and colleagues reported that the irregular pulsation detected by 4D-CTA may potentially lead to a morphologic change in shape at follow-up and had a significant correlation with dark reddish thinner walls [40]. Therefore, it indicates that this method may be useful for identifying aneurysms with a higher risk of rupture.

Our study has some limitations: first of all, there is a lack of comparison with the results of surgery in terms of morphology, as a previous study showed a comparison with DSA could be sufficient to evaluate the morphology of aneurysms [41]. Secondly, the occurrence of thrombosis in the aneurysm wasn’t evaluated due to the absence of thrombosis in all aneurysms in this study. However, it is critical for the treatment plan decision-making whether or not thrombosis is present. Finally, the relatively small sample size may limit the power of the analysis. Hence, future prospective studies with larger patient populations and longer follow-up period are needed to further explore the role of 4D-CTA in the visualization of aneurysmal pulsation.

## Conclusions

Even considering the limitations of our preliminary results due to the relatively restricted group of patients enrolled and the short follow-up, our data show the reliability of 4D-CTA with ECG-gated reconstructions in defining the morphological characteristics and dynamic pulsation of the aneurysm wall. Moreover, the optimal correlation between the findings provided by the 4D-CTA and the intraoperative observations a supports possible role of this technique in identifying unruptured aneurysms that are at high risk of rupture. On the basis of findings obtained with 4D-CTA, we could obtain novel, highly useful information regarding as-yet unruptured aneurysms. Our purpose for the future is to recruit more patients and conduct longer follow-up periods, looking for further confirmations of our data. We intend to undertake such studies at the earliest opportunity.

### Supplementary Information

**Additional file 1.**

**Additional file 2.**

**Additional file 3.**

**Additional file 4.**

**Additional file 5.**

## Data Availability

The datasets generated during and analyzed during the current study are not publicly available due to patient privacy concerns but are available from the corresponding author on reasonable request.

## Abbreviations

4D-CTA: Four-Dimensional Computed Tomography Angiography
3D-CTA: Three-Dimensional Computed Tomography Angiography
ECG: Electrocardiogram-Gated
UIAs: Unruptured Intracranial Aneurysms
DSA: Digital Subtraction Angiography
SAH: Subarachnoid Hemorrhage
DLP: Dose-Length Product
CTDI vol: Volume CT Dose Index
MRA: Magnetic Resonance Angiography
FOV: Field-Of-View
